# Human Alpha Defensin 5 Expression in the Human Kidney and Urinary Tract

**DOI:** 10.1371/journal.pone.0031712

**Published:** 2012-02-16

**Authors:** John David Spencer, David S. Hains, Edith Porter, Charles L. Bevins, Julianne DiRosario, Brian Becknell, Huanyu Wang, Andrew L. Schwaderer

**Affiliations:** 1 Pediatric Nephrology Fellowship Program, Nationwide Children's Hospital, Columbus, Ohio, United States of America; 2 Division of Nephrology, Department of Pediatrics, Nationwide Children's Hospital, Columbus, Ohio, United States of America; 3 Center for Clinical and Translational Research, The Research Institute at Nationwide Children's Hospital, Columbus, Ohio, United States of America; 4 Department of Biological Sciences, California State University Los Angeles, Los Angeles, California, United States of America; 5 Department of Microbiology and Immunology, School of Medicine, University of California Davis, Davis, California, United States of America; Universidad Nacional de La Plata., United States of America

## Abstract

**Background:**

The mechanisms that maintain sterility in the urinary tract are incompletely understood. Recent studies have implicated the importance of antimicrobial peptides (AMP) in protecting the urinary tract from infection. Here, we characterize the expression and relevance of the AMP human alpha-defensin 5 (HD5) in the human kidney and urinary tract in normal and infected subjects.

**Methodology/Principal Findings:**

Using RNA isolated from human kidney, ureter, and bladder tissue, we performed quantitative real-time PCR to show that *DEFA5*, the gene encoding HD5, is constitutively expressed throughout the urinary tract. With pyelonephritis, *DEFA5* expression significantly increased in the kidney. Using immunoblot analysis, HD5 production also increased with pyelonephritis. Immunostaining localized HD5 to the urothelium of the bladder and ureter. In the kidney, HD5 was primarily produced in the distal nephron and collecting tubules. Using immunoblot and ELISA assays, HD5 was not routinely detected in non-infected human urine samples while mean urinary HD5 production increased with *E.coli* urinary tract infection.

**Conclusions/Significance:**

*DEFA5* is expressed throughout the urinary tract in non-infected subjects. Specifically, HD5 is expressed throughout the urothelium of the lower urinary tract and in the collecting tubules of the kidney. With infection, HD5 expression increases in the kidney and levels become detectable in the urine. To our knowledge, our findings represent the first to quantitate HD5 expression and production in the human kidney. Moreover, this is the first report to detect the presence of HD5 in infected urine samples. Our results suggest that HD5 may have an important role in maintaining urinary tract sterility.

## Introduction

The urinary tract, apart from the urethral meatus, is usually sterile despite its proximity with fecal flora. The precise mechanisms by which the urinary tract maintains its sterility are not well understood. Proposed mechanisms contributing to defense of the urinary tract include urine flow, alterations in urine pH and osmolarity, regular bladder emptying, chemical-defense components of the uroepithelium, and epithelial shedding/influx of effector immune cells with bacterial stimulation [1]. In addition, antimicrobial peptides (AMPs) have recently been shown to have an important role in maintaining urinary tract sterility [2].

AMPs, which serve as natural antibiotics produced by nearly all organisms, are a ubiquitous component of the innate immune system. AMPs are cationic molecules expressed by phagocytic white cells and epithelial cells. In humans and other mammals, defensins are a major family of AMPs. Defensins typically have broad-spectrum antimicrobial activity against gram-positive and gram-negative bacteria, viruses, fungi, and protozoa [2], [3]. Defensins are initially synthesized as preproproteins and undergo processing to become mature, biologically active peptides [4]. In humans, defensins are classified into one of two families depending on their disulfide bridging pattern – the alpha-defensins or the beta-defensins [5].

In the urinary tract, the beta-defensins are widely expressed throughout the uroepithelium. Epithelial cells lining the kidney's loop of Henle, distal tubule, and collecting duct constitutively express human beta-defensin 1 (hBD1). Although urinary levels of hBD1 are insufficient to kill invading bacteria, hBD1 provides a fast-acting antimicrobial coating of tubular lumens and prevents infection by inhibiting bacterial attachment to the urothelium [6]. Recent studies indicate that the redox-state of hBD1 significantly affects antimicrobial potency, such that the reduced peptide is much more potent that the disulfide-linked oxidized form [7]. The significance of this at the urothelial surface has not been determined. Another defensin, human beta-defensin 2 (hBD2) is not constitutively expressed in healthy kidney tissue; however, hBD2 expression is induced by infection [8].

Unlike the beta-defensins, the role of epithelial-derived alpha-defensins is not well described in the urinary tract. The expression and function of alpha-defensin HD5 and HD6 have mostly been reported in the small intestine where they are secreted by Paneth cells into the intestinal crypts and contribute to the balance of intestinal microbiotica [9]. HD5 has also been localized in the male and female genital tracts, with evidence suggesting that it is inducible and important in eradicating infection [10], [11], [12]. Urinary HD5 has been detected in patients who have undergone ileal neobladder reconstruction and ileal conduit urinary diversion whereby the source of HD5 production was primarily credited to the ileal Paneth cells [13], [14]. HD5 has antibacterial activity against common uropathogenic gram-positive bacteria and gram-negative bacteria [15]. HD5 also has antimicrobial activity against uropathogenic viruses like adenovirus and BK virus [16], [17], [18]. In this study, we provide the initial description and quantification of HD5 expression in the human kidney and further define its expression in the lower urinary tract during sterility and infection.

## Results

### *DEFA5* mRNA is constitutively expressed in human bladder, ureter, and kidney

**Figure 1:**
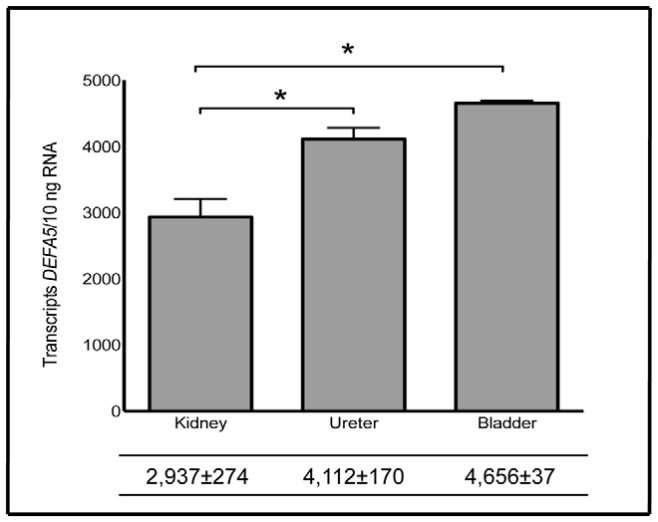
Expression of *DEFA5* in human kidney, ureter, and bladder. *DEFA5* mRNA transcript levels were quantified by real-time PCR in non-infected kidney, ureter, bladder. Shown are the results of three independent samples. In the table below, the mean transcript levels are shown with the SEM. *DEFA5* expression was significantly greater in the lower urinary tract (*p* = 0.014).

Quantitative real-time PCR demonstrates that all tested bladder, ureter, and kidney specimens without infection constitutively expressed *DEFA5*. *DEFA5* expression was significantly greater in the lower urinary tract than the upper urinary tract (*p* = 0.014). In the bladder (*n* = 4), mean *DEFA5* expression was 4,656±37 transcripts per 10 ng RNA and in the ureter (*n* = 4) mean *DEFA5* expression was 4,112±170 transcripts per 10 ng RNA (Figure 1A). In the kidney (*n* = 6), mean *DEFA5* expression was 2,937±274 transcripts per 10 ng RNA. *DEFA5* expression was analyzed separately in the renal cortex, medulla, and pelvis. *DEFA5* expression did not significantly vary by kidney region (*p* = 0.45).

### *DEFA5* expression and HD5 peptide expression increase with pyelonephritis

**Figure 2:**
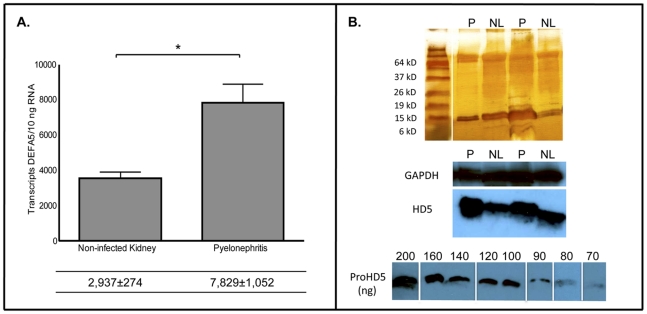
HD5 expression increases with pyelonephritis. (**A**) *DEFA5* mRNA transcript levels were quantified by real-time PCR in non-infected kidney tissue and in kidney tissue with pyelonephritis. Shown are the results for three independent samples. In the table below, the mean transcript levels are shown with the SEM. *DEFA5* expression was significantly greater with pyelonephritis (*p* = 0.019). (**B**) To confirm the increase in message is accompanied by an increase in HD5 protein production, cationic peptides from the same non-infected kidney tissues (NL) and kidney tissue with pyelonephritis (P) were subjected to SDS PAGE followed by Western immunoblot analysis. Each lane contained the equivalent of 800 µg of cationic protein. Silver stained PAGE gels (top panel) confirmed equal protein loading into each lane. Immunoblot analysis for GAPDH and HD5 (middle panel). Serial dilutions of proHD5 (200 ng–70 ng) were subjected to SDS PAGE followed by Western immunoblot analysis (bottom panel).

Quantitative real-time PCR analysis performed on kidney tissues with pyelonephritis demonstrated a significant increase in *DEFA5* expression compared to non-infected kidney tissues. With pyelonephritis (*n* = 6), mean *DEFA5* expression increased to 7,829±1,052 transcripts per 10 ng RNA (*p* = 0.019) (Figure 2A).

To further evaluate this increase in expression with pyelonephritis, we performed immunoblot analysis on the same kidney tissue used for real-time PCR analysis to evaluate for concurrent increases in HD5 peptide production (Figure 2B, middle panel). Immunoblot analysis, using polyclonal HD5 antisera, demonstrated significantly greater HD5 peptide production in kidney tissues with pyelonephritis (*n* = 6) compared to non-infected kidney tissues (*n* = 6). Non-infected kidney tissues expressed 300±25 ng HD5/gram wet tissue weight while kidney tissue with pyelonephritis expressed 600±21 ng HD5/gram wet tissue weight (*p*<0.0001). Blots were re-probed with GAPDH to serve as a loading control (Figure 2B, middle panel) and a silver stain was also performed to confirm equal protein loading (Figure 2B, top panel).

### HD5 peptide is produced throughout the human kidney and urinary tract

**Figure 3:**
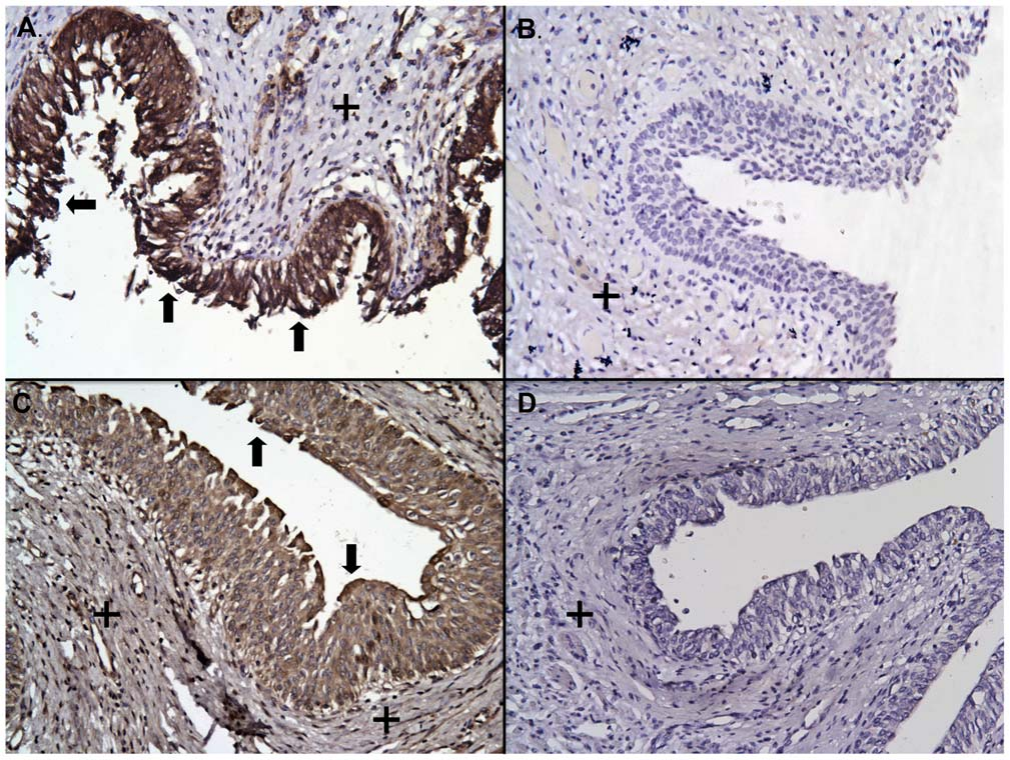
HD5 is expressed throughout the urothelium of the ureter and bladder. Immunohistochemistry demonstrates HD5 expression (brown/arrows) throughout the urothelium of the human bladder (**A**) and ureter (**C**). Immunostaining was most prominent in the luminal surfaces (brown/arrows). Immunostaining was not detected in the smooth muscle layers of the bladder or ureter (+). Negative control bladder (**B**) and ureter (**D**) showed no immunostaining. Magnification 20×.

**Figure 4:**
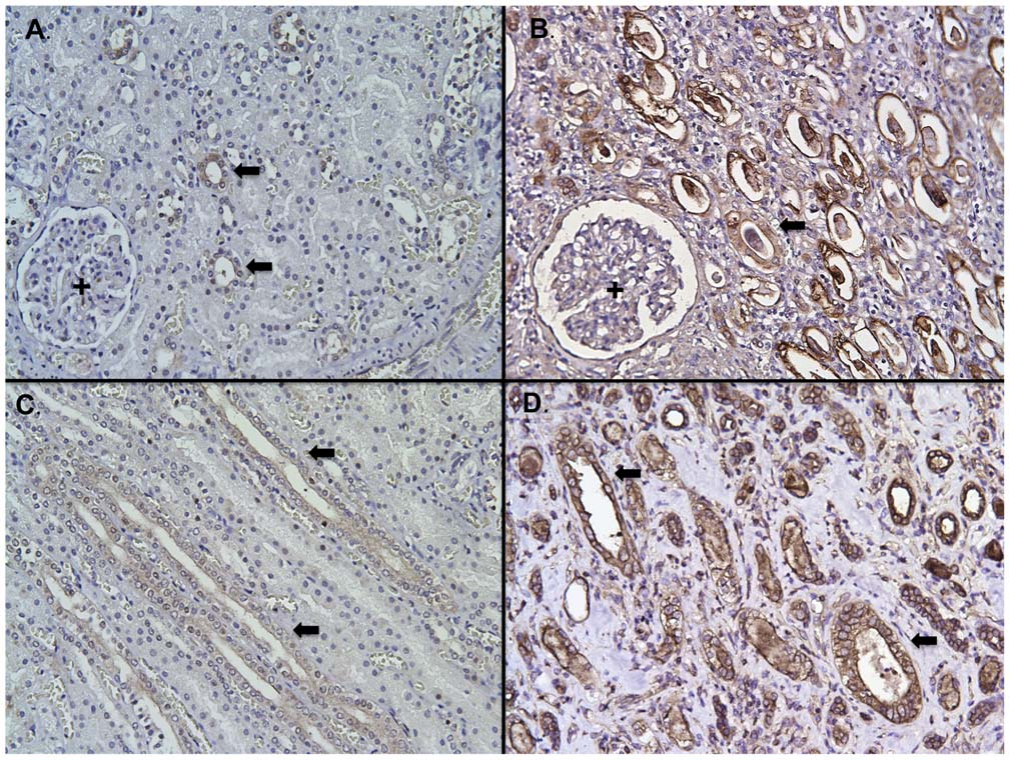
HD5 production in non-infected human kidney and kidney with pyelonephritis. Immunohistochemistry demonstrates HD5 production in isolated renal tubules (brown/arrows) in non-infected renal cortex (**A**) and medulla (**C**). With pyelonephritis, HD5 production increased in the renal tubules of the cortex (**B**) and medulla (**D**). The glomeruli (+) show no immunostaining in non-infected kidney samples and with pyelonephritis. Negative controls showed no immunostaining (not shown). Magnification 20×.

**Figure 5:**
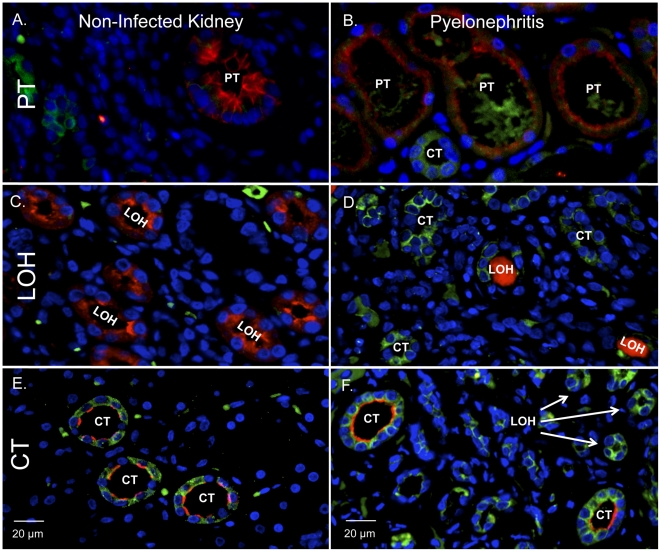
Tubular HD5 expression in states of sterility and infection. Human kidney labeled for HD5 (green), nuclei (blue) and nephron specific markers (red). Segment markers consisted of AQP-2 for collecting tubules (CT), THP for the loop of Henle (LOH), and AQP-1 for proximal tubules (PT). **A/B**: HD5 (green) was produced in the collecting tubules (red apical AQP-2 staining) of non-infected kidney tissue (A) and with pyelonephritis (B). Arrows indicate HD5 is produced in other nephron segments. **C/D**: HD5 (green) was expressed in the loops of Henle (red THP staining) in non-infected kidney tissue (C) and with pyelonephritis (D). **E/F**: HD5 (green) shows minimal production in the proximal tubules (red AQP-1 staining) of non-infected kidney tissue (E) and with pyelonephritis (F). Magnification 40×.

Immunostaining was performed to localize HD5 production in the urinary tract. Immunohistochemistry (IHC) showed that HD5 immunoreactivity was present throughout the urothelium of the ureter and bladder of all investigated specimens (*n* = 4, Figure 3). IHC also showed that HD5 was produced in tubules of the renal cortex and medulla of all specimens (*n* = 6, Figure 4). Immunofluorescence (IF) demonstrated that HD5 expression was greatest in the distal nephron and the collecting tubules (Figure 5). The interstitium and glomeruli did not show HD5 immunoreactivity. The immunostaining shown in Figures 3, 4, and 5 was performed using a mouse monoclonal anti-HD5 antibody (8C8) that recognizes the HD5 propeptide (Abcam, Cambridge, MA). Results were similar when using rabbit polyclonal anti-HD5 antibody that recognizes the HD5 propeptide and mature peptide (data not shown) – suggesting that HD5 is stored as a propeptide.

With pyelonephritis, HD5 immunostaining markedly increased. HD5 immunoreactivity increased throughout the proximal nephron, the distal nephron, and the collecting tubules (Figure 4 and Figure 5). As in non-infected specimens, the glomeruli and interstitium showed no HD5 expression with infection. Negative controls showed no HD5 immunoreactivity.

### Infected human urine contains measurable concentrations of HD5 peptide

**Figure 6:**
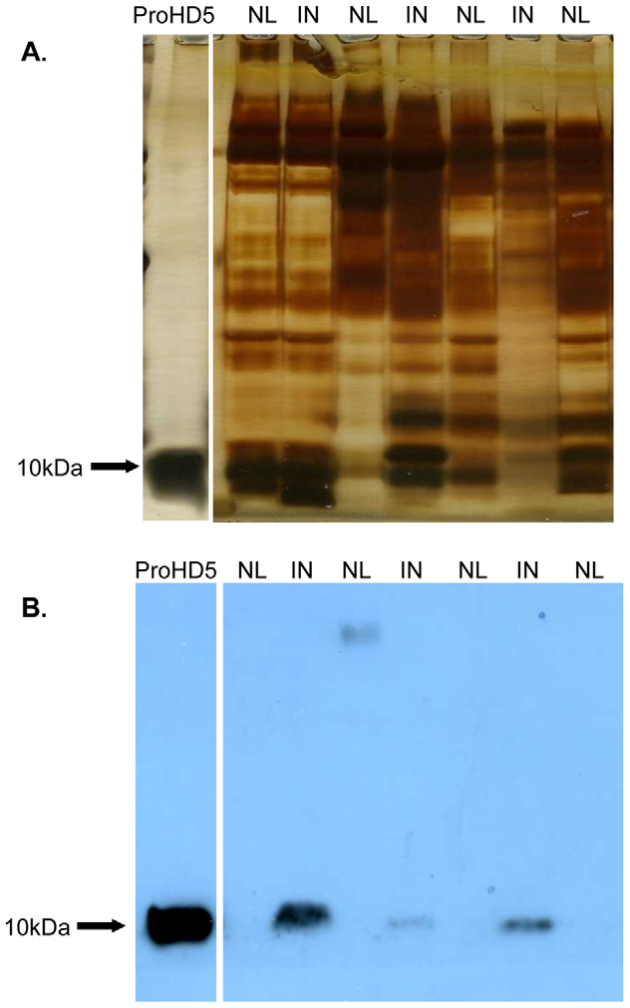
HD5 is present in infected urine samples. HD5 levels in sterile urine samples (n = 15) and urine samples infected with uropathogenic *E.coli* (*n* = 15). Cationic peptides from non-infected urine (NL) and infected urine (IN) were subjected to SDS PAGE followed by Western immunoblot analysis. Each lane contained the equivalent of 300 µg of urinary cationic protein. (**A**) Silver stained PAGE gels with 150 ng ProHD5 as standard to confirm equal protein loading into each lane. (**B**) Western blot analysis with 200 ng ProHD5 as standard.

**Figure 7:**
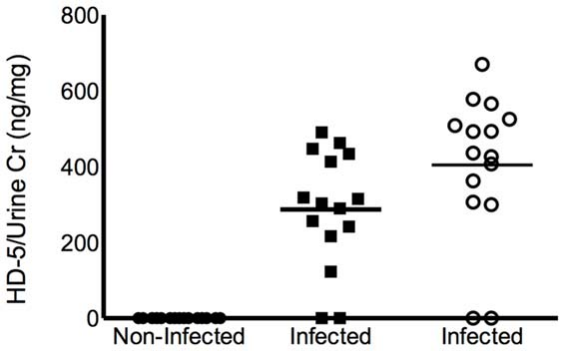
Urinary production of HD5 in infected urine samples. HD5 levels in sterile urine samples and urine samples infected with uropathogenic *E.coli*. The square boxes depict urinary proHD5 standardized to urine creatinine detected by ELISA assay using monoclonal antibody 8C8. The open circles depict urinary proHD5 and mature HD5 standardized to urine creatinine by immunoblot analysis. ProHD5 and mature HD5 were not detected in sterile urine samples using polyclonal HD5 antisera.

Immunoblot analysis, using rabbit polyclonal antiserum that detects the precursor proHD5 and further processed forms identified measurable levels of HD5 in 13 of the 15 tested urine samples infected with uropathogenic *E.coli* (Figure 6A and B). When present, HD5 levels, normalized to urine creatinine, ranged from 299.8–669.7±30 ng HD5/mg urine Cr (110.67 ng/mL–276.67 ng/mL), which corresponds to 11.10–27.67 nmol/L. In non-infected urine samples (*n* = 15), HD5 was at or below the detection limit of (<50 ng). Enzyme linked immunosorbant assay (ELISA) on the same urine samples, using the mouse monoclonal antibody (8C8) that detects only the precursor proHD5, detected measurable levels of proHD5 in 13 of the 15 infected samples tested. Urinary proHD5 concentrations ranged from 122.78–490.0±0.03 ng HD5/mg urine Cr (30.02 ng/mL–200.5 ng/mL) when present (Figure 7).

## Discussion

In this study, we provide the initial characterization HD5 in the human kidney, ureter, and bladder. HD5 has been shown to be an important AMP that prevents invasive infection in the intestine and is up-regulated in the genital tract during infection [11], [18], [19], [20], [21]. Epithelial derived AMPs, like HD5, have been shown to be important in maintaining sterility in the urinary tract [8], [22], [23], [24]. Deficiencies in these innate mucosal defenses may result in acute and/or chronic infection [25], [26].

Our quantitative real-time PCR results demonstrate that *DEFA5* is constitutively expressed in human kidney, ureter, and bladder. *DEFA5* expression increases from the upper urinary tract to the lower urinary tract – following the flow of the urinary stream and the increasing closeness to the microbiota's point of invasion. To our knowledge, this is the first study to quantify *DEFA5* expression in the human kidney and bladder. Recently, Townes *et al* demonstrated that normal distal ureter can express *DEFA5* [14]. Our results also suggest that *DEFA5* is constitutively expressed by the distal ureter. Although *DEFA5* expression in the urinary tract is nearly 100-fold lower than that found in gastrointestinal tissues, basal uroepithelial expression of *DEFA5* is comparable to the expression of previously described urinary tract AMPs like cathelicidin, hBD-1, hBD-2, and ribonuclease 7 [6], [8], [22], [24], [27].

In contrast to the mature gastrointestinal tract where *DEFA5* is expressed at high levels in both states of health and infection/inflammation, our quantitative real-time PCR data demonstrate that *DEFA5* expression in the kidney is significantly up-regulated with infection [27], [28], [29]. This inducible pattern of expression is analogous to *DEFA5* expression in the female reproductive tract where *DEFA5* expression increases with salpingitis [30]. In the urinary tract, Townes *et al* have shown a trend toward greater ureteral expression of *DEFA5* in patients with UTIs who have undergone ileal conduit urinary diversion [14].

Our immunoblot analysis complements the real-time PCR data by demonstrating that HD5 peptide production increases with pyelonephritis. This inducible pattern of HD5 production is similar to that seen in some members of the beta-defensin family. hBD2 shows an inducible pattern of production with infection of the urinary tract and/or chronic pyelonephritis [8], [31], [32]. HD5 peptide production has been shown to increase in the upper female genital tract and male urethra with inflammation [11], [30].

Using immunostaining, we demonstrate that HD5 is uniformly produced throughout the urothelium of the ureter and bladder. Because the vast majority of UTIs result from fecal flora that ascend into the bladder, these results suggest that HD5 expression is present in locations where microbial exposure occurs most frequently [33]. Therefore, HD5 is ideally positioned to prevent an ascending microbial infection. In the kidney, HD5 is primarily produced in the distal nephron and collecting tubule. These findings suggest that HD5 is produced in locations where it will be positioned to have optimal antimicrobial activity. Previous studies have shown that the antimicrobial properties of defensins are dependent on salt concentration – with higher salt concentrations decreasing antimicrobial activity [23], [34], [35]. We speculate that the low salt concentrations of the distal nephron and collecting tubule provide a favorable milieu for HD5 antimicrobial activity.

Immunoblot and ELISA analysis also demonstrates that HD5 is secreted into the urine at low levels. Given the size (8.1 kDa and 3.7 kDa) and positive charge of proHD5 and fully processed HD5, it is possible that some urinary HD5 peptide originates, at least in part, from plasma filtrate. Yet, there is little evidence suggesting that HD5 persists in the plasma [27]. Additionally, to appear in the urine, HD5 would need to escape the efficient peptide absorption mechanisms in the proximal tubule [6], [36]. Finally, the urine samples used underwent centrifugation before the assays were performed, removing cellular sources of HD5.

At the detected concentrations, urinary HD5 is unlikely to be directly antimicrobial against uropathogenic microorganisms as fully processed HD5 has a minimal inhibitory concentration (MIC) between 6–10 µg/mL against common gram-negative uropathogenic bacteria [15]. However, mucosal surface concentrations are likely to be higher. Furthermore, immunoblot analysis demonstrates that HD5 concentrations are significantly greater in the kidney. These results suggest that HD5 is involved in mucosal defense of the urinary tract. An analogous pattern is seen with hBD1, where urinary concentrations of hBD1 are insufficient to exhibit antimicrobial activity but higher peptide concentrations are detected near the renal tubules [6], [31].

Because IHC demonstrates that proHD5 is a major form of HD5 in the kidney and because both mature and proHD5 are detected in the urine, we speculate that HD5 is primarily secreted as a precursor molecule and then undergoes proteolytic processing to generate mature forms – similar to the gastrointestinal and genitourinary tracts [11], [30], [37]. In the small intestine, Paneth cells produce trypsin, which cleaves the HD5 propeptide so that the fully processed peptide is the predominant form in the intestinal lumen [37]. In the male urethra, the key processing and activating enzymes for HD5 are neutrophil proteases contributed by neutrophils recruited to the site of infection [11]. The contributions of epithelial trypsinogen, urinary trypsin, and/or neutrophil proteases on HD5 processing in the urinary tract during infection remain to be determined. Moreover, the effects of changes in the urinary environment on HD5 function (alterations in osmolarity, pH, and cationic concentrations) also remain to be determined.

In conclusion, this is the first study to identify and quantitate the expression and production of HD5 in the urinary tract. Our results suggest that HD5 is an epithelial-derived AMP that may play an important role in the innate immunity of the human uroepithelium preventing the translocation of invading pathogens into the circulation. Elucidation of the factors that regulate HD5 production may lend novel insight into the pathogenesis of UTIs in patients at risk for UTIs and patients with chronic infections.

## Methods

### Study approval

Informed written consent was obtained from all patients participating in this study. For subjects less than 18 year of age, written parental/guardian consent was obtained. The Nationwide Children's Hospital (NCH) Institutional Review Board approved this study along with the consent process and documents (IRB07-00383).

### Human Tissue and Urine Samples

Non-infected distal ureter and bladder tissue (*n* = 4) was obtained from children undergoing ureteral re-implantation for reasons other than recurrent infection. Non-infected kidney samples (*n* = 6) were obtained from patients undergoing nephrectomy for renal tumors. Tissue samples were free of microscopic signs of disease or inflammation. Kidney tissue from patients with chronic pyelonephritis was provided by the Cooperative Human Tissue Network (*n* = 6) [38]. Two independent pathologists confirmed the histopathologic diagnosis of pyelonephritis. Tissue samples were snap-frozen or preserved as neutral formalin-fixed paraffin-embedded sections. Sections of non-infected kidney tissue were dissected into cortex, medulla, or renal pelvis before storage (*n* = 4).

Non-infected and infected urines samples were obtained from children presenting to the NCH emergency department or the nephrology clinic. The diagnosis of a UTI was made by a positive urine culture according to the American Academy of Pediatrics Guidelines [39]. All infected urine samples had >10^6^ CFU/mL of *E.coli*. Urinary pH values ranged from 5.5 to 8.5 (mean urine pH 6.9). Urinary ionic composition was not measured. Urine samples were centrifuged to remove urine sediment, and protease inhibitor cocktail was added (Thermo Scientific, Rockford, IL, USA).

### Ribonucleic Acid Isolation and Reverse Transcription

Total RNA was isolated from frozen tissue using the Promega Total RNA Isolation System (Promega, Madison, WI, USA). For cDNA synthesis, 4–8 µg of total RNA was reverse transcribed with Superscript III reverse transcriptase using an oligo-(dT)_12–18_ primer according to the supplier's protocol (Invitrogen, Carlsbad, CA, USA).

### Cloning of Gene Specific Plasmids for Standard Curves

The cDNA encoding *DEFA5* and *GAPDH* were cloned into a 4-Topo plasmid vector (Invitrogen) according to manufacturer's instructions. Plasmids were sequenced to confirm that the correct constructs were obtained. Serial dilutions of gene specific plasmids were quantitated (by spectrophotometric absorbance at 260 nm and ethidium bromide staining agarose gel electrophoresis) and used in real-time PCR to generate standard curves for each reaction.

### Quantitative Real-time PCR

Quantitative real-time PCR was performed using single-stranded cDNA from human kidney, ureter, and bladder tissue with specific oligonucleotide primer pairs using the 7500 Real-Time PCR System (Applied Biosystems, Carlsbad, CA, USA). PCR exon junction spanning primers were designed and sequences were confirmed using DNAstar® Laser Gene SeqBuilder (*DEFA5* forward primer: 5′- TCC CTC CTG CAG GTG ACC CCA-3′ and *DEFA5* reverse primer 5′-GTG GCT CTT GCC TGA GAA CCT GA-3′).

Briefly, cDNA corresponding to 10 ng RNA served as a template in a 25 µl reaction containing 2.0 µM of each primer and 1× Light-Cycler-Fast Start DNA Master SYBR Green mix. The PCR conditions were: initial denaturation at 95°C for 10 minutes, followed by 40 cycles with each cycle consisting of denaturation at 94°C for 30 seconds, annealing at 64°C for 30 seconds, and extension at 72°C for 30 seconds. The cycle-to-cycle fluorescence emission was monitored at 530 nm and analyzed using 7500 Software V2.0.3 (Applied Biosystems). Gene specific plasmid standards were included with every set of reactions. As a positive control, terminal ileum RNA was included with each set of reactions and results were compared to previously published standards [27].

To confirm PCR amplification of the intended product, a representative sample was analyzed by electrophoresis on a 1.5% agarose gel. The products were visualized by ethidium bromide staining and compared to DNA size standards to confirm anticipated product size (not shown). In addition, a melting temperature profile curve of every PCR reaction was determined at the end of each reaction.

### HD5 Antibodies

The HD5 propeptide (AA20-94) and partially processed forms (AA36-94 and 56–94) were identified using a commercially available mouse monoclonal (8C8) anti-HD5 antibody (Abcam). The HD5 propeptide and the mature peptide (AA63-94) were identified using previously described rabbit polyclonal antiserum [13], [40].

### Immunoblot

Sections of the same kidney specimens used for quantitative real time PCR analysis (3–6 mg wet weight) were pulverized using a mortar and pestle in liquid nitrogen and dissolved in RIPA buffer with protease inhibitors. Urinary proteins were extracted from urine samples using the Proteospin™ Urine Protein Concentration Micro Kit (Norgen Biotek Corporation, Thorold, ON, Canada). Kidney and urine protein concentrations were quantified using a modified Bradford Assay and confirmed using an OD_280 nm_ reading. Equal concentrations of kidney tissue or urinary protein were loaded onto 18% sodium dodecyl sulfate gradient gels and subjected to electrophoresis. To ensure equal protein loading, a silver stain was performed.

After electrophoretic separation, proteins were transferred onto a nitrocellulose membrane. After fixation with 0.05% glutaraldehyde in TBS, the membranes were blocked in 5% fat-free milk for 30 to 60 minutes and incubated in a 1∶1,000 dilution of rabbit polyclonal HD5 antiserum overnight. After washing, the secondary antibody, an anti-rabbit horseradish peroxidase-conjugated anti-rabbit IgG diluted 1∶10,000 (Cell Signaling Technology, Danvers, MA, USA), was applied for 1 hour at room temperature. Immunoblots from kidney tissues were also probed with anti-GAPDH antibody (Sigma Aldrich) for two hours at room temperature and then incubated with the secondary antibody described above. The proteins were visualized using and ECL detection system and chemiluminescence film according to the manufacturer's instructions (BioExpress, Kaysville, UT, USA).

HD5 was quantitated by comparing resulting band intensities with a serial dilution of recombinant standard proHD5 protein (Peptides International, Louisville, KY, USA). Kidney HD5 concentrations were standardized to wet tissue weight. Urinary HD 5 concentrations were divided by urine creatinine to establish standardized urine HD5-to-creatinine ratios (µg/mg) to account for urine dilution. Urine creatinine concentrations were determined using the Oxford Biomedical Research creatinine microplate assay (Rochester Hills, Michigan, USA).

### Immunohistochemistry

Following deparaffinization, rehydration, and antigen retrieval, a biotin block and a serum-free protein block were performed (Superblock, ScyTek Laboratories, Logan, UT, USA). The slides were incubated overnight at 4°C with monoclonal mouse HD5 (8C8) antibody (1∶200; Abcam) or rabbit polyclonal antiserum (1∶500) followed by anti-polyvalent biotinylated antibody and UltraTek Streptavidin/HRP (ScyTek Laboratories). Sections were developed using 0.1% diaminobenzidine tetrachloride with 0.02% hydrogen peroxide and counterstained with hematoxylin. Negative controls sections were incubated with non-immune serum in place of HD5 antibody.

### Immunofluorescence

Double-labeled immunofluorescence was performed to help localize HD5 expression in the kidney. The collecting duct was double-labeled for principal cells with goat polyclonal anti-human aquaporin-2 antibody (Santa Cruz Biotechnology, Santa Cruz, CA, USA) [41]. The loop of Henle was double-labeled with mouse polyclonal anti-human uromodulin antibody (Sigma-Aldrich) and the proximal tubule was double-labeled with goat polyclonal antihuman aquaporin-1 (Santa Cruz) [41], [42]. Rhodamine donkey polyclonal anti-goat (Jackson ImmunoResearch Laboratories, West Grove, PA, USA), rhodamine goat anti-mouse (Jackson ImmunoResearch Laboratories), and FITC donkey polyclonal anti-rabbit (Santa Cruz) served as the secondary antibodies.

All sections were prepared as outlined above. They were incubated with a mixture of mouse antisera against HD5 (1∶200) (Abcam), AQP-2 (1∶500), uromodulin (1∶500), AQP-1 (1∶400) at room temperature for 90 minutes. The secondary antibody was applied for 90 minutes at room temperature and the sections were mounted using mounting media with DAPI. Non-immune serum was used as a negative control. The slides were examined with a Leica DM4000B microscope and digitally photographed using Spot RT camera/software (Diagnostic Instruments, Sterling Heights, MI, USA).

### ELISA

96-well flat-bottomed plates (Maxisorb, Nunc™, Rochester, NY, USA) were coated overnight at 4°C with mouse monoclonal antibody to HD5 (3 µg/mL) (Abcam). After blocking with synthetic blocking buffer (Kem-En-Tec Diagnostics, Denmark), 100 µL standards and/or urine samples were added to the wells and incubated for 2 hours at room temperature. Serial dilutions of recombinant HD5 protein served as the standards (Novus Biologicals). Following incubation with a biotinylated (Lightning-Link Biotin Antibody Labeling Kit, Novus Biologicals) mouse monoclonal antibody for 2 hours at room temperature, streptavidin-horse radish peroxidase (Biolegend, San Diego CA, USA) was added for 30 minutes. After incubation with TMB substrate solution for 15 minutes (Kem-En-Tec Diagnostics), the reaction was terminated with STOP solution (Cell Signaling Technology, Danvers, MA, USA) and read at a wavelength of 450 nm. HD5 concentrations from the ELISA assay were divided by urine creatinine to establish standardized urine HD5-to-creatinine ratios (µg/mg) to account for urine dilution as described above.
